# m^6^A Methyltransferase METTL3 Promotes the Progression of Primary Acral Melanoma *via* Mediating TXNDC5 Methylation

**DOI:** 10.3389/fonc.2021.770325

**Published:** 2022-01-18

**Authors:** Zhanghui Yue, Meng Cao, Anlan Hong, Qian Zhang, Guoqiang Zhang, Zhibin Jin, Liang Zhao, Qiang Wang, Fang Fang, Yan Wang, Jianfang Sun

**Affiliations:** ^1^ Institute of Dermatology, Chinese Academy of Medical Sciences and Peking Union Medical College, Nanjing, China; ^2^ Department of Dermatology, The First Hospital of Hebei Medical University, Shijiazhuang, China; ^3^ Department of Ultrasound, Nanjing Drum Tower Hospital, Nanjing, China

**Keywords:** Mettl3 (methyltransferase like 3), melanoma, TXNDC5, m6A (N^6^-methyladenose), progression

## Abstract

m^6^A modification is one of the most important post-transcriptional modifications in RNA and plays an important role in promoting translation or decay of RNAs. The role of m^6^A modifications has been highlighted by increasing evidence in various cancers, which, however, is rarely explored in acral melanoma. Here, we demonstrated that m^6^A level was highly elevated in acral melanoma tissues, along with the expression of METTL3, one of the most important m^6^A methyltransferase. Besides, higher expression of METTL3 messenger RNA (mRNA) correlated with a higher stage in primary acral melanoma patients. Knockdown of METTL3 decreased global m^6^A level in melanoma cells. Furthermore, METTL3 knockdown suppressed the proliferation, migration, and invasion of melanoma cells. In METTL3 knockdown xenograft mouse models, we observed decreased volumes and weights of melanoma tissues. Mechanistically, we found that METTL3 regulates certain m^6^A-methylated transcripts, thioredoxin domain containing protein 5 (TXNDC5), with the confirmation of RNA-seq, MeRIP-seq, and Western blot. These data suggest that METTL3 may play a key role in the progression of acral melanoma, and targeting the m^6^A dependent-METTL3 signaling pathway may serve as a promising therapeutic strategy for management of patients of acral melanomas.

## Introduction

Melanoma represents the most invasive skin cancer type and comprises the most skin cancer-related deaths (1). It has distinct clinicopathological subtypes with biological heterogeneity (2). Acral melanoma (AM), defined as melanoma arising from the glabrous skin of palms, soles, and nail beds, is a rare subtype in Caucasians but accounting for more than 70% in Asian population (3). The previous study revealed that the progression of AM has distinct clinical and genetic features, potentially due to diagnosis at a more advanced clinical stage, or due to biological differences favoring tumor aggression, AM usually has poorer prognosis than other types of cutaneous melanoma (4–6). In recent years, targeted therapies and checkpoint immunotherapies have greatly improved the prognosis of advanced melanomas (7, 8). However, more than half of Asian patients are unable to benefit from immunotherapy and targeted therapy (9), which might attribute to lower tumor mutation burden, programmed-death 1 ligand low expression, phenotype plasticity, etc. (10–13). Up to date, most AM patients do not have successful genotype-specific treatment options. Consequently, determining the molecular mechanisms underlying AM progression is critical for improving therapy and diagnosis. The multistep process involves complicated interplays between epigenetics and transcriptomic alterations results in the AM progression. From a clinical perspective, trauma (14), mechanical stress (15, 16), and some other epigenetic stimulus seemed to be associated with the risk of AM develop. Meanwhile, increasing evidence has revealed that AM has greater prevalence of epigenetic alternation than genetic changes (17–19). Hence, targeting epigenetic regulation represents a novel approach for AM treatment.

N6-methyladenosine (m^6^A), the most prevalent eukaryotic RNA modification, recently emerged as a major post-transcriptional modulator of gene expression (20). It influences the multifaceted process of RNA metabolism, including splicing, stability, and translation efficiency. Dynamic m^6^A modification participates in diverse important life processes (21). Accumulating evidence in recent years reveals that aberrant m^6^A RNA modifications levels and dysregulation of m^6^A regulators underlie various human cancers, including glioblastoma (22), hepatocellular carcinoma (23), cervical cancer (24), leukemia (25), and breast cancer (26). M^6^A occurs primarily in the consensus RRACH motif (R ¼ GorA; H ¼ A, C, or U) and is dynamically regulated by the “writer,” “eraser,” and “reader” proteins (27, 28). Writers mainly belong to the m^6^A-METTL-associated complex and include METTL3, METTL14, WTAP, VIRMA, etc. The demethylases FTO and ALKBH5 function as erasers, removing N‐methyl from adenosine (29). The m^6^A sites are specifically recognized by “Readers,” including YTH family, elF3, and IGF2BP family proteins (30). METTL3 is the core catalytic component and cooperates with other proteins to methylate adenosine. The onset and progression of various cancers are affected by METTL3, either dependent or independent on its m^6^A RNA methyltransferase activity (31–33). METTL3 principally functions as an oncogene to promote carcinogenesis in various cancers: METTL3 depletion induced cell differentiation and apoptosis to delay acute myeloid leukemia progression by regulating m^6^A modification on MYC, BCL2, and PTEN mRNAs (25); high expression of METTL3 correlates with poor clinical outcomes in glioblastoma, and the m^6^A modification on SRSF transcript added by METTL3 leads to human glioblastoma outgrowth and self-renewal (34). In bladder cancer, METTL3 was upregulated in patient samples, and it benefits cancer progression by mediating several m^6^A modification targets (CDCP1, ITGA6, AFF4, IKBKB, RELA, and MYC), in which YTH family served as the binding protein of the target transcripts (35–37). METTL3 also exhibits tumor suppressor function in some cases: lower expression of METTL3 was detected in renal cell carcinoma patients and predicts a favorable prognosis (38). Nevertheless, not much is known about the biological and pathological role of METTL3-dependent m^6^A modification in acral melanoma.

In this study, we found that the imbalance of global m^6^A abundance and dysregulated expressions of m^6^A regulators were frequent in primary acral melanoma patients. The high expression of METTL3 was positively related to advanced clinical stage in primary acral melanoma. We then confirmed that METTL3 functions as an oncoprotein in primary acral melanoma by both *in vitro* and *in vivo* experiments. Further molecular mechanism investigation indicated that TXNDC5 was downregulated by decreasing m^6^A-levels after METTL3 knockdown and ultimately inhibiting the progression of primary acral melanoma. Thus, our findings extend the understanding of m^6^A-driven machinery in acral melanoma progression and proposed that the METTL3-m^6^A-TXNDC5 axis may serve as biomarker and therapeutic strategy in the clinic.

## Materials and Methods

### Cell Culture and Transfection

The normal human epidermal melanocyte (HEMa) was extracted from fresh foreskin tissue donated after circumcision in adults and cultured in melanocyte medium with 5% fetal bovine serum (Sciencell, USA). A875, MV3 cell lines were kindly supplied by Prof. Xiulian Xu, who worked in our institute. HMY-1 cell line was a gift from Prof. Yan Kong who worked in Peking University Cancer Hospital and Institute. A375, SK-MEL-28, and A2058 were obtained from the Cell Bank of Type Culture Collection (Guangzhou Cellcook Biotech Co., Ltd., Guangzhou, China). A375 and SK-MEL-28 were derived from cutaneous melanoma, A875 was derived from melanoma brain metastasis tissue, A2058 and MV3 were derived from metastatic lymph node, and HMY-1 was derived from acral melanoma. Except for HMY-1 that originate from Asians, the other cell lines originate from Caucasians. Human melanoma cell lines were cultured in Dulbecco’s modified Eagle’s medium (A375, A875, A2058, MV3, and HMY-1) or minimal essential medium (SK-MEL-28) supplemented with 10% fetal bovine serum (BioInd, Beit HaEmek, Israel) and penicillin-streptomycin solution (Gibco, Grand Island, NY, USA) at 37°C in a 5% CO_2_ atmosphere. All cell lines were routinely tested for mycoplasma contamination and identified by sequence tandem repeat profiling. Short hairpin RNA (shRNA) sequences were designed by Hanbio Biotechnology Co., Ltd. (Shanghai, China) to target human METTL3. After annealing, double strands of short hairpin RNA (shRNA) were inserted into the lentiviral pHBLV-U6-MCS-CMV-ZsGreen-PGK-PURO vector (Hanbio), named shMETTL3, and the non-targeted control was named NTC. Stable cell lines were established after selection with 1 µg/ml puromycin (Millipore, Billerica, MA, USA). The target gene knockdown efficiency was evaluated *via* quantitative RT-PCR (qRT-PCR) and Western blot analysis.

### Patients and Specimens

This study was approved by the Ethics Committee of the Institute of Dermatology at the Chinese Academy of Medical Sciences and carried out according to the Declaration of Helsinki. Thirty-four pairs of fresh melanoma tumor tissues and adjacent normal specimens were obtained from patients who underwent surgical resection without any preoperative treatment between September 2017 and July 2019. All samples were confirmed by pathological examination according to the American Joint Committee on Cancer (AJCC) Cancer Staging Manual (8^th^ edition). The clinicopathological features of all patients are summarized in **Table 1**. Written informed consent for the biological studies was obtained from each patient.

**Table 1:** Correlation between METTL3 expression and clinicopathologic features of acral melanoma patients (n = 34).

Feature	Number	METTL3 Expression		p-value
		High	Low	
Age (years)				1.000
≥60	22	11	11	
<60	12	6	6	
Gender				0.481
Male	13	8	5	
Female	21	9	12	
Ulceration				0.438
With	9	6	3	
Without	25	11	14	
Breslow thickness				0.014*
≤1.0 mm	14	4	10	
1.0–2.0 mm	7	2	5	
2.0–4.0 mm	9	7	2	
≥4.0 mm	4	4	0	
AJCC clinical staging^a^				0.044*
0	12	3	9	
I	6	2	4	
II	13	10	3	
III	3	2	1	
Primary site				0.181
Hand	8	2	6	
Foot	23	14	9	
Nail bed	3	1	2	

a No TNM IV stage melanoma patients was included.

*p < 0.05 indicates a significant relationship among the variables.

### Protein Isolation and Western Blotting Analysis

Cells and tissues were lysed using 1× radioimmunoprecipitation assay (RIPA) buffer (9806, Cell Signaling Technology, Danvers, MA, USA) containing protease inhibitors (Complete; Roche, Basel, Switzerland) and phosphatase inhibitors (PhosSTOP; Roche). Equal amounts of proteins were loaded and separated by 10% sodium dodecyl sulfate–polyacrylamide gel electrophoresis (SDS-PAGE), transferred onto polyvinylidene fluoride membranes (Immobile P; Millipore), and detected by immunoblotting with enhanced chemiluminescence (Chemidoc XRS, Bio-Rad, Hercules, CA, USA). Antibodies used for Western blotting were as follows unless otherwise specified: METTL3 (Abcam, Cambridge, UK; ab195352, 1:1,000) and TXNDC5 (Proteintech, China, 19834-1-AP, 1:10,000); glyceraldehyde 3-phosphate dehydrogenase (GAPDH) (Abcam, ab181602, 1:10,000) was used as a loading control.

### Wound-Healing Assay

A total of 2 × 10^4^ cells/well were seeded into a six-well plate. Upon cells reaching 90% confluence, a wound-like gap was made in the cell monolayer using a 200-µl pipette tip. Cells were washed with phosphate-buffered saline three times and incubated for the indicated time. The migration distance of cells at 0, 24, and 48 h was photographed and analyzed using ImageJ software (NIH, Bethesda, MD, USA).

### Migration and Invasion Analysis

Cell migration and invasion assays were carried out in a 24-well Transwell chamber (Corning, NY, USA) inserts with an 8-μm pore polycarbonate filter. For the migration assays, approximately 5 × 10^4^ cells were suspended in 200 μl serum-free Dulbecco’s modified Eagle’s medium and added to the upper chamber. The lower chamber contained 600 μl complete culture medium with 10% fetal bovine serum. After 24–48 h, the cells were fixed in 4% paraformaldehyde, followed by staining with crystal violet. Cells on the upper surface of the membrane were removed with a cotton swab. Five fields per chamber were selected and photographed randomly using an inverted microscope (Nikon, Tokyo, Japan). For the invasion assay, 100 μl diluted Matrigel (Corning, NY, USA) was dispensed into the upper chamber before seeding the cells, and the remaining process was performed similarly in the migration assay. ImageJ software was used to count the migrated and invaded cells.

### RNA Isolation and Quantitative Real-Time PCR

We performed qRT-PCR to measure the gene expression levels in AM tissues and cell lines. Total RNA from different samples was extracted using TRIzol reagent (Invitrogen) according to the manufacturer’s instructions. The purity and concentration of RNA were determined using a NanoDrop 2000 spectrometer (Thermo Fisher Scientific, Waltham, MA, USA). RNA samples (500–1,000 ng) were subjected to RT-PCR using the TaKaRa RT-PCR kit (Takara, Shiga, Japan). Expression levels were quantified by qPCR in a LightCycler ^®^ 480 Instrument II device (Roche Applied Science, Mannheim, Germany) using SYBR Master Mix (without ROX Vazyme, Nanjing, China). All primers were provided by General Biotech Co., Ltd. (Shanghai, China). Target gene expression was normalized to the housekeeping gene GAPDH using the ΔCt method, with a relative expression equal to 2^−ΔCt^. All sequences of the primers used in our study were obtained from primerbank (https://pga.mgh.harvard.edu/primerbank/), and the specificity of the primers were verified by the primer blast function of NCBI. Primers sequences used in this study are listed in **Supplementary Table S1**.

### Immunohistochemistry and Antibodies

Tissues were previously fixed in 10% formalin, paraffin embedded, and cut into 4-µm sections. The slides were subjected to dewaxing, rehydration, antigen retrieval, and blocking and then incubated with primary antibody [anti-METTL3 (1:1,000, Abcam) and anti-Ki-67 (1:400, Abcam)] overnight at 4°C. On the following day, after washing with phosphate-buffered saline, the slides were further incubated for 1 h with the secondary antibody at room temperature. Diaminobenzidine and hematoxylin were used to visualize the reaction and counterstain the slide.

### Cell Proliferation Assay and Colony-Formation Assays

For the cell proliferation analysis, 2 × 10^4^ cells were seeded into 12-well plates at 72 h after transfection; green fluorescent protein (GFP)-positive cells were counted on four consecutive days using a countess automated cell counter (Invitrogen). Cell Counting Kit-8 (Dojindo, Kumamoto, Japan) was used to determine the effect of METTL3 on cell viability; the transfected cells were cultured in the 96‐well plate at a density of 4 × 10^3^ cells/well for 4 days. CCK-8 dye solution was added to each well at a certain time point. Following 2 h of incubation at 37°C, the absorbance was measured on a microplate reader (Thermo Fisher Scientific) at 450 nm.

The colony-formation assay was performed using a six-well plate. A total of 600 cells/well transfected cells were incubated for 2 weeks. The colonies were fixed in 4% paraformaldehyde and stained with 1% crystal violet for 30 min.

### 5-Ethynyl-20-Deoxyuridine Assay

5-Ethynyl-20-deoxyuridine (EdU) Cell Proliferation Kit with Alexa Fluor 555 (Epizyme, Shanghai, China) was used to detect DNA synthesis and cell proliferation. Cells were incubated at 37°C with Dulbecco’s modified Eagle’s medium (DMEM) containing 10 μM EdU for 2 h. After being fixed with 4% paraformaldehyde, these cells were subsequently stained with Azide 555 Dye Solution and Hoechst 33342. An inverted fluorescent microscope was used to capture three randomly selected fields to visualize the EdU-stained cells.

### Global m^6^A Quantification

Total RNA isolation was performed to measure global changes in the m^6^A modification level. The content of m^6^A was determined using an EpiQuik m^6^A RNA Methylation Quantification Kit (cat. P-9005, EpiGentek, Farmingdale, NY, USA) following the manufacturer’s protocol. Briefly, negative and diluted positive control RNA, and 200 ng total RNA, were used to analyze each sample.

### m^6^A Dot Blot

The dot blot assay was conducted according to the bio-protocol database (https://en.bio-protocol.org/e2095). Briefly, the indicated amount of total RNA was denatured and spotted onto a Hybond-N+ membrane (GE Healthcare, Little Chalfont, UK) and crosslinked using a UV crosslinker. The membrane was first stained with methylene blue (MB) for 2 h and then washed with RNase-free water for 1 h. Images were acquired using an Epiwhite light as the loading control. After MB was washed with RNase-free water, the membrane was incubated in 5% bovine serum albumin/Tris-buffered saline containing 1% Tween-20 as the blocking buffer and then with an anti-m^6^A antibody (1:1,000; Abcam) overnight at 4°C. Thereafter, the membrane was washed, incubated with secondary antibody, and washed again. Finally, the membrane was exposed to Hyperfilm ECL, and images were acquired. The m^6^A signal was quantified by TotalLab software (Amersham Pharmacia Biotech, Amersham, UK) and normalized to MB levels.

### *In Vivo* Xenograft Experiment

Sixteen BALB/c nude mice (male, 6-week-old) were raised under pathogen-free conditions and randomly divided into two groups. A total of 2 × 10^6^ A375 NTC or sh-METTL3#2 cells were subcutaneously inoculated into the right hind flank. Body weight and tumor size were measured every other day. The tumors were harvested at the end of the observation period, and tumor weight and gross images were recorded (11 days after inoculation). The tumors were embedded in RNA later and 10% formalin for further detection. Animals were treated humanely, and all procedures complied with the National Institutes of Health (NIH) Guide for the Care and Use of Laboratory Animals or equivalent guidelines and approved by the Institutional Animal Care and Use Committee of our hospital.

### RNA-Seq and m^6^A-RNA Immunoprecipitation Sequencing

The RNA amount and purity of each sample were quantified using a NanoDrop ND-1000 (Wilmington, DE, USA). RNA integrity was assessed with a Bioanalyzer 2100 (Agilent Technologies, Santa Clara, CA, USA) with RNA integrity number >7.0 and confirmed by electrophoresis in a denaturing agarose gel. Approximately 25 μg of total RNA representing a specific adipose type was used to deplete ribosomal RNA according to the manuscript of the Epicentre Ribo-Zero Gold Kit (Illumina, San Diego, CA, USA). Following purification, the ribosomal-depleted RNA was fragmented into small pieces using the Magnesium RNA Fragmentation Module (cat. e6150, New England Biolabs, Ipswich, MA, USA) at 86°C for 7 min. The cleaved RNA fragments were incubated for 2 h at 4°C with an m^6^A-specific antibody (No. 202003, Synaptic Systems, Goettingen, Germany) in IP buffer (50 mM Tris–HCl, 750 mM NaCl, and 0.5% Igepal CA-630). The immunoprecipitated RNA was reverse-transcribed to prepare the complementary DNA (cDNA) using SuperScript™ II Reverse Transcriptase (cat. 1896649, Invitrogen), which was used to synthesize U-labeled second-stranded DNAs with *Escherichia coli* DNA polymerase I (cat. m0209, New England Biolabs), RNase H (cat. m0297, New England Biolabs), and dUTP solution (cat. R0133, Thermo Fisher Scientific). An A-base was then added to the blunt ends of each strand to prepare them for ligation to the indexed adapters. Each adapter contained a T-base overhang for ligating the adapter to the A-tailed fragmented DNA. Single- or dual-index adapters were ligated to the fragments, and size selection was performed with AMPureXP beads. After treatment with the heat-labile UDG enzyme (cat. m0280, New England Biolabs) of the U-labeled second-stranded DNAs, the ligated products were amplified with PCR under the following conditions: initial denaturation at 95°C for 3 min; 8 cycles of denaturation at 98°C for 15 s, annealing at 60°C for 15 s, and extension at 72°C for 30 s; with a final extension at 72°C for 5 min. The average insert size of the final cDNA library was 300 ± 50 bp. Finally, 2 × 150-bp paired-end sequencing (PE150) was performed on an Illumina Novaseq™ 6000 (LC-Bio Technology Co., Ltd., Hangzhou, China) following the vendor’s recommended protocol.

### Statistical Analysis

Bioinformatic analysis including Gene Ontology (GO) and Kyoto Encyclopedia of Genes and Genomes (KEGG) enrichment was performed using OmicStudio tools (https://www.omicstudio.cn/tool). Transcript expression levels were dichotomized based on the median expression, and a false discovery rate <0.05 indicated statistical significance. All statistical analyses were performed using SPSS version 22.0 software (SPSS, Inc., Chicago IL, USA). The relationship between METTL3 expression and clinicopathological patient characteristics was analyzed using Pearson χ^2^ test or Fisher exact test. Individual groups were compared by two-tailed Student’s t-test, and one-way analysis of variance was used for multiple groups. Pearson correlation analysis was used to estimate the relationship between the expression level of METTL3 and TXNDC5. Data are presented as the mean ± SD from at least three biological replicates, with *p < 0.05, **p < 0.01, ***p < 0.001, and ****p < 0. 0001 indicating the significance levels.

## Results

### Abnormal RNA m^6^A Modification Level and m^6^A-Related Enzyme Expression in Melanoma

**Figure 1:**
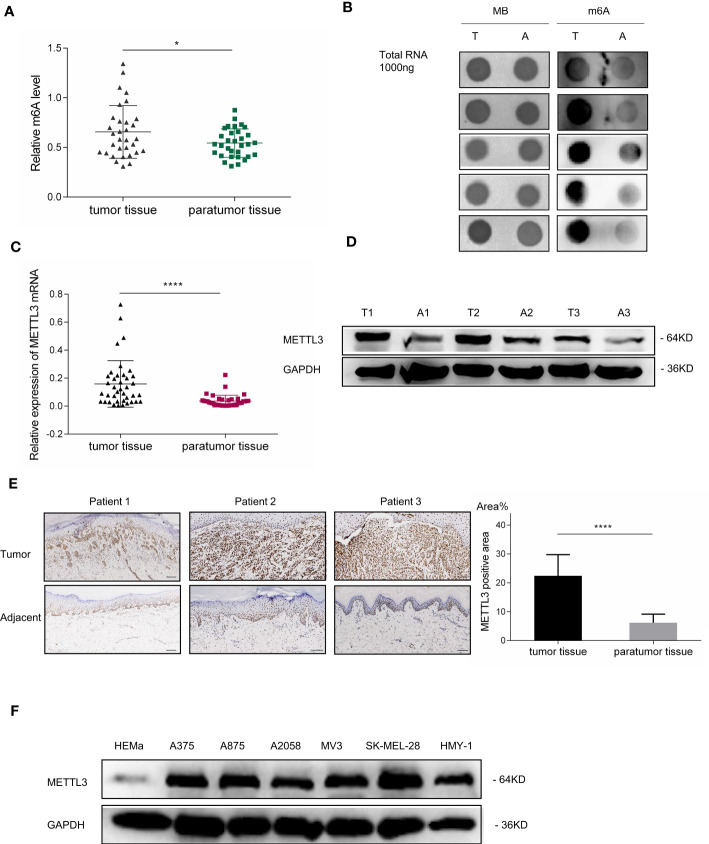
m^6^A RNA methylation level and METTL3 expression in patients with melanoma. **(A)** Global m^6^A quantification of total RNA from 32 melanoma specimens demonstrated the elevation of m^6^A levels in tumor tissues; scatterplot displays statistical results. Bar charts show the mean ± SD of each group (n = 32). **(B)** Dot blot of total RNA extract from five pairs of melanoma tumor tissues and adjacent normal tissues stained with an anti-m^6^A antibody. Methylene blue represents loading control of RNA samples. **(C)** METTL3 expression in melanoma tumor and adjacent normal tissues determined *via* qRT- PCR. Bar charts show the mean ± SD of each group (n = 34). **(D)** Western blotting confirmed METTL3 upregulation in melanoma. GAPDH served as a loading control. **(E)** IHC revealed masses of METTL3-positive cells in AM tumor tissues. Relatively negative staining was observed in adjacent normal tissues. Bar = 100 μm. Bar chart shows the mean ± SD of each group (n = 12). **(F)** Western blot analysis indicated that METTL3 protein levels of HEMa were lower in melanoma cells. Data are presented as the mean ± SD from at least three biological replicates, with *p < 0.05, and ****p < 0.0001 indicating the significance levels.

Given that the imbalance of m^6^A modification exists in various cancers, the m^6^A level in melanoma remains uncertain. To determine the potential involvement of m^6^A modifications in melanoma, we first examined m^6^A levels in 32 pairs of melanoma tumor and adjacent normal tissues. The m^6^A RNA Methylation Quantification assay showed that m^6^A modification levels were remarkably elevated in acral melanoma tumor tissues compared with the adjacent normal tissues (**Figure 1A**, p < 0.05), suggesting that m^6^A alternations play an indispensable role in the carcinogenesis of melanoma. These results were confirmed in the dot blot assay (**Figure 1B**).

The RNA m^6^A modification is dynamically modulated by both methyltransferases and demethyltransferases. We hypothesized that the upregulation of m^6^A modification was caused by the dysregulation of m^6^A-related genes in melanoma, the expressions of which were analyzed by RT-PCR in 34 pairs of melanoma tumor and adjacent normal tissues. Results showed that the mRNA expression of METTL3, the core m^6^A methyltransferase, was significantly upregulated (**Figure 1C**, p < 0.0001), which may be responsible for the upregulation of global m^6^A levels in melanoma (**Table 1**).

To further examine METTL3 expression in melanoma, the baseline characteristics of 34 patients with melanoma were considered and are summarized in **Table 1**, and it was found that the high expression of METTL3 mRNA was related to advanced clinical stages and reflected the clinical significance of METTL3 in melanoma. The increased METTL3 protein levels in tumor tissue were detected by Western blotting (**Figure 1D**). Immunohistochemistry (IHC) also determined the upregulated protein expression of METTL3 in 12/12 (100%) melanoma tissue samples relative to the corresponding adjacent normal tissues (**Figure 1E**). In melanoma cell lines, METTL3 was significantly highly expressed at both the mRNA and protein level compared to that in normal human epidermal melanocytes (HEMa) (**Supplementary Figure S1A** and **Figure 1F**).

### METTL3 Silencing Decreases Global m^6^A Level in Melanoma Cell Lines

**Figure 2:**
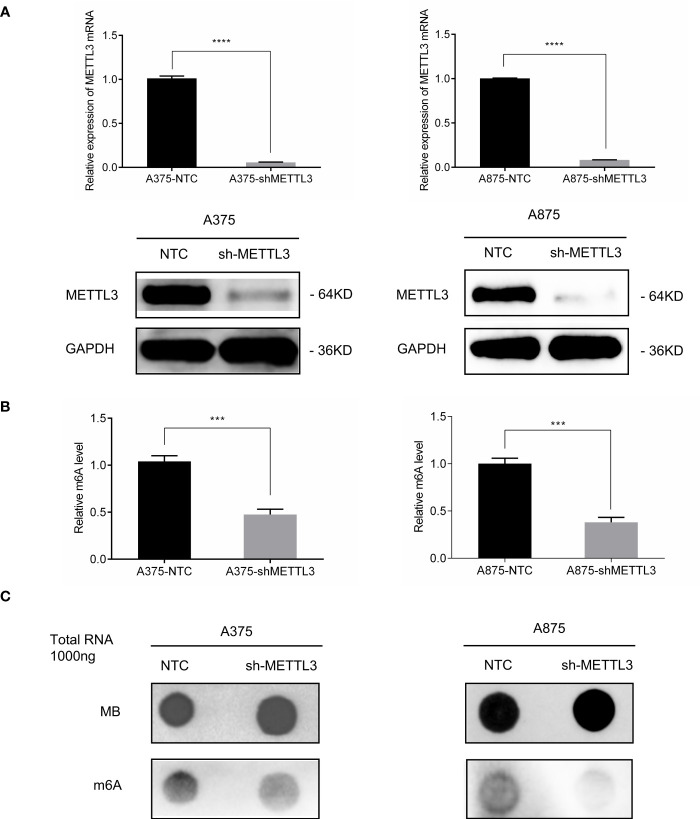
METTL3 silencing decreased global m^6^A level in melanoma cell lines. **(A)** METTL3 mRNA and protein levels showing knockdown efficiency in A375 and A875; bar chart showing data from three independent experiments; mean ± SD of each group. **(B)** m^6^A modification levels were decreased both in A375-shMETTL3 and A875-shMETTL3 as determined *via* colorimetric ELISA. **(C)** Dot blots showing relative total RNA m^6^A (bottom) and loading control (top). Data are presented as the mean ± SD. ***p < 0.001,****p < 0.0001.

To explore the function of METTL3 in melanoma, we silenced the expression of METTL3 in A375 and A875 cells with shRNA transfection, which reduced METTL3 expression by approximately 90% relative to that in non-targeted control (NTC) cells, as confirmed by qRT-PCR and Western blotting (**Figure 2A**). Then, global m^6^A quantification and m^6^A dot blot assays were performed and demonstrated that m^6^A modification level was downregulated heavily by METTL3 silencing in A375 and A875 cells (**Figures 2B, C**).

### METTL3 Knockdown Inhibits Melanoma Cell Proliferation, Migration, and Invasion *In Vitro*

**Figure 3:**
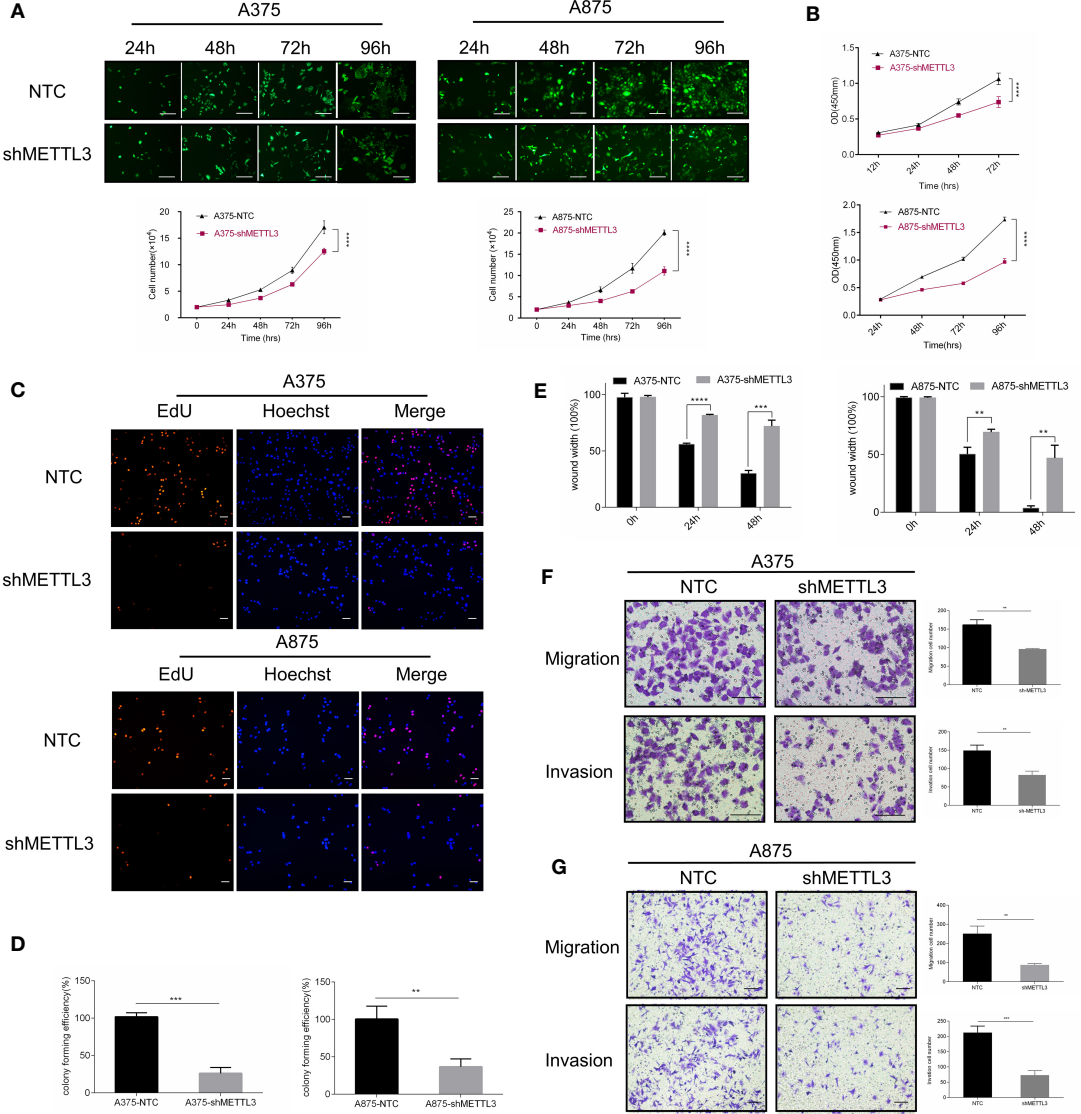
METTL3 knockdown inhibits melanoma cell proliferation, migration, and invasion *in vitro*. **(A)** Continuous cell count of A375 and A875 transfected cells with GFP reflected cell proliferation ability. **(B)** Cell proliferation assay by CCK8 method. **(C)** EdU cell proliferation, EdU (red), Hoechst (blue), scale bar = 100 μm. **(D)** Quantification of the results from colony-formation assay. **(E)** The statistical histogram of wound-healing assays in A375 and A875 transfected cells. Transwell assays with or without Matrigel indicated suppressed migration and invasion of **(F)** A375 and **(G)** A875 cells following METTL3 knockdown. Bar = 100 μm. Mean ± SD of each group (n = 3). Significant differences were evaluated using two-way or one-way ANOVA. **P < 0.01, ***P < 0.001, ****P < 0.0001.

We next assessed the changes in cellular behaviors in A375 and A875 cells caused by METTL3 knockdown. Cell counting, CCK-8 assays were carried out and indicated that the knockdown of METTL3 expression significantly inhibited melanoma cell proliferation (**Figures 3A, B** and **Supplementary Figure S2A**). In EdU assays, the number of EdU-positive cells exhibited the decreased tendency in A375/A875 shMETTL3 groups compared to that of NTC groups (**Figure 3C**), and the ratio of EdU-positive cells is shown in **Supplementary Figure S2**. Moreover, a similar trend was noted when carrying out in colony formation assays. For A375 and A875 cells, the colony forming rates of NTC cells were 3.9- and 2.7-fold higher, respectively, than those of shMETTL3 cells (**Figure 3D**), which meant that METTL3 knockdown inhibited melanoma cell proliferation and colony formation ability *in vitro*. As high expression of METTL3 correlated with advanced stage of melanoma, which was revealed above, we speculated that the upregulation of METTL3 might be involved in migration and invasion of melanoma cells. We therefore compared the migration and invasion abilities of NTC and shMETTL3 cells. Wound healing assays and Transwell analysis indicated that METTL3 knockdown significantly impaired the abilities of migration and invasion in melanoma cells. (**Figures 3E**–**G** and **Supplementary Figure S2C**).

### METTL3 Knockdown Suppresses Melanoma Tumor Growth *In Vivo*

**Figure 4:**
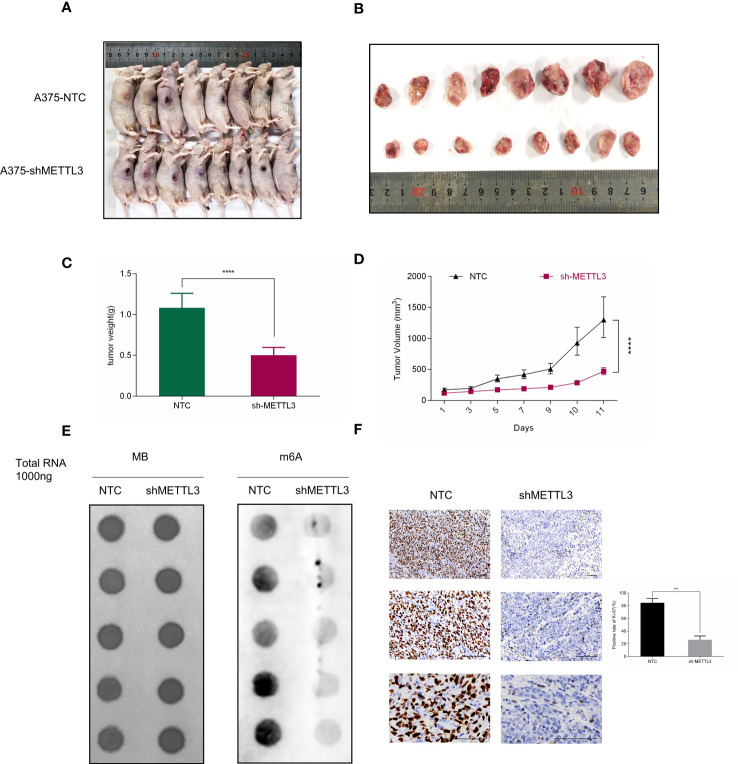
METTL3 depletion delays tumor growth in nude mice. **(A, B)** Representative photographs and tumor size are shown at 11 days after inoculation. **(C)** Xenografts derived from A375-shMETTL3 cells demonstrated **(C)** tumor weight and **(D)** volume that were much lower than their A375 NTC cell-derived counterparts. **(E)** m^6^A level of METLL3 knockdown group xenografts was much lower than in control tumors. **(F)** Representative Ki^6^7 staining of xenograft tumor section and quantification of Ki^6^7-positive cells are shown. Bar = 100 μm. Data are presented as the mean ± SD (n = 5). Significant differences were evaluated using Student t-test. ***P < 0.001, ****P < 0.0001.

To confirm the functional role of METTL3 *in vivo*, A375 shMETTL3 or NTC cells were subcutaneously inoculated into the right flank of nude mice, respectively (**Figure 4A**). Eleven days later, the xenograft tumors were collected to measure the tumor volume and weight, which were considerably lower in the shMETTL3 group than the in NTC group, as **Figures 4B**–**D** show. IHC was performed subsequently and showed that the expression of Ki-67, the proliferation marker in melanoma, decreased correspondingly in the sh-METTL3 group (**Figure 4F**). Then, RNA was extracted from xenograft tumors, and global m^6^A levels were detected, which were significantly lower as well in the sh-METTL3 group than in the NTC group (**Figure 4E**).

### METTL3 Knockdown Dysregulates Gene Expression

**Figure 5:**
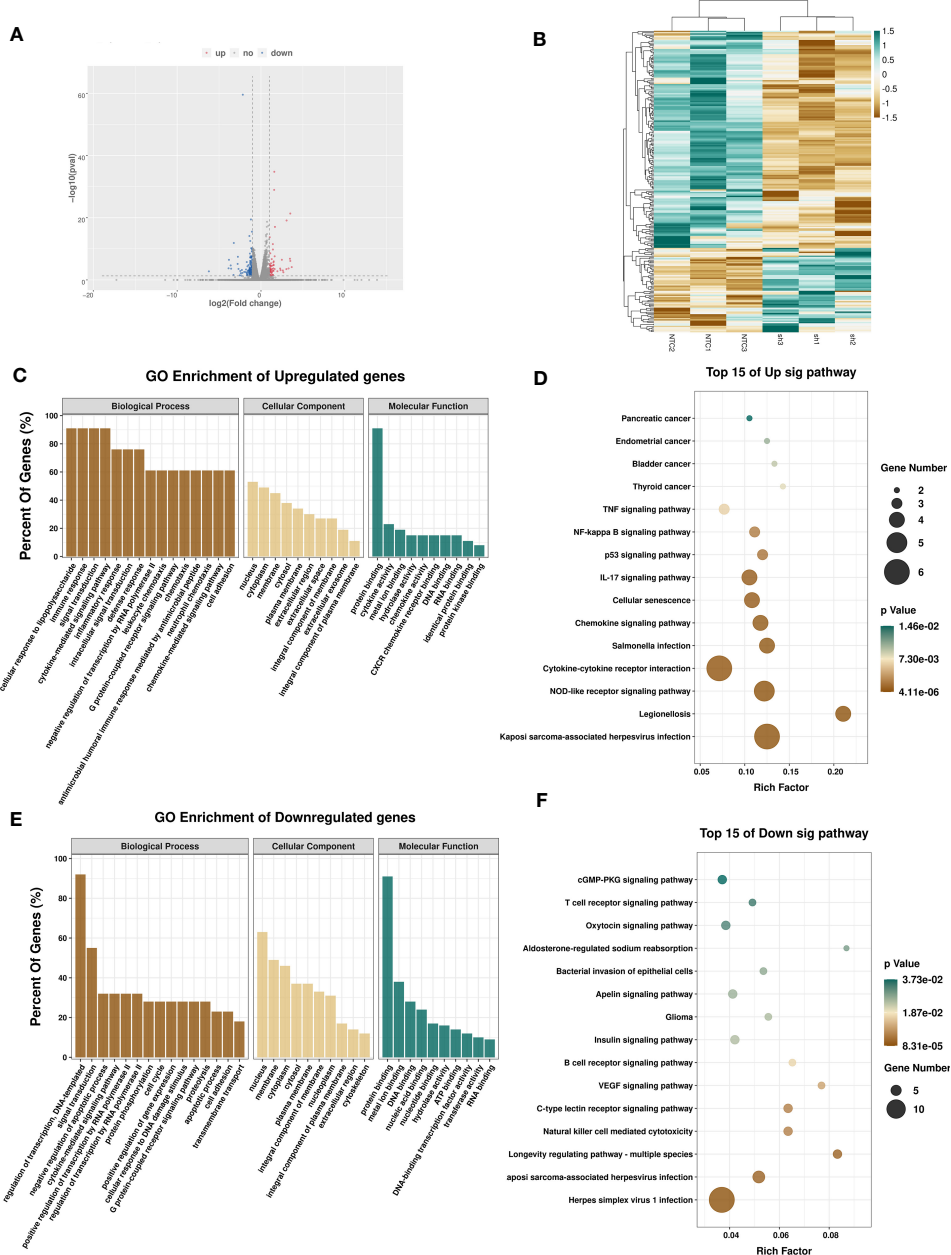
Effects of METTL3 on transcription profile of melanoma cells. **(A)** Volcano plot showing significantly upregulated (red) and downregulated (blue) genes between A375-NTC (NTC) and A375-shMETTL3(sh) groups. **(B)** Heatmap showing differentially expressed transcripts between NTC and sh groups. GO and KEGG analyses of significantly upregulated **(C, D)** and downregulated **(E, F)** genes identified *via* RNA-seq suggesting the involvement of DEGs in signal transduction.

To determine the regulatory role of METTL3 in gene expression, we performed RNA-seq in A375 shMETTL3 and NTC cells. A total of 33,013 genes transcripts were identified, and the expression of these transcripts was quantified. Upon comparison with NTC cells, 232 significantly differentially expressed genes (DEGs) were identified in METTL3-depleted A375 cells (sh) (**Figure 5A**). Nearly 72% (167 of 232) of these genes were downregulated regardless of m^6^A modifications (fold-change ≥2 and p < 0.05) (**Figure 5B**). Furthermore, Gene Ontology (GO) analysis revealed that DEGs involved in the immune response, cell adhesion, and inflammatory response were significantly upregulated after METTL3 depletion (**Figure 5C**). Genes downregulated in sh-METTL3 cells were related to GO terms associated with cell cycle and negative regulation of apoptotic process (**Figure 5E**). KEGG pathway enrichment associated upregulated genes with the cytokine receptor interaction, NOD-like receptor, P53, and nuclear factor kappa B (NF-κB) signaling pathway, which were closely related to the tumor micro-environment, cell cycle, and growth. Pathways enriched for downregulated genes included the cGMP-PKG signaling pathway, insulin pathway, natural killer cell-mediated cytotoxicity, and HSV-1 infection (**Figures 5D, F**). Taken together, METTL3 exhibited cancer-promoting effects based on transcriptional regulation in melanoma.

### METTL3 Acts Through m^6^A Modification

**Figure 6:**
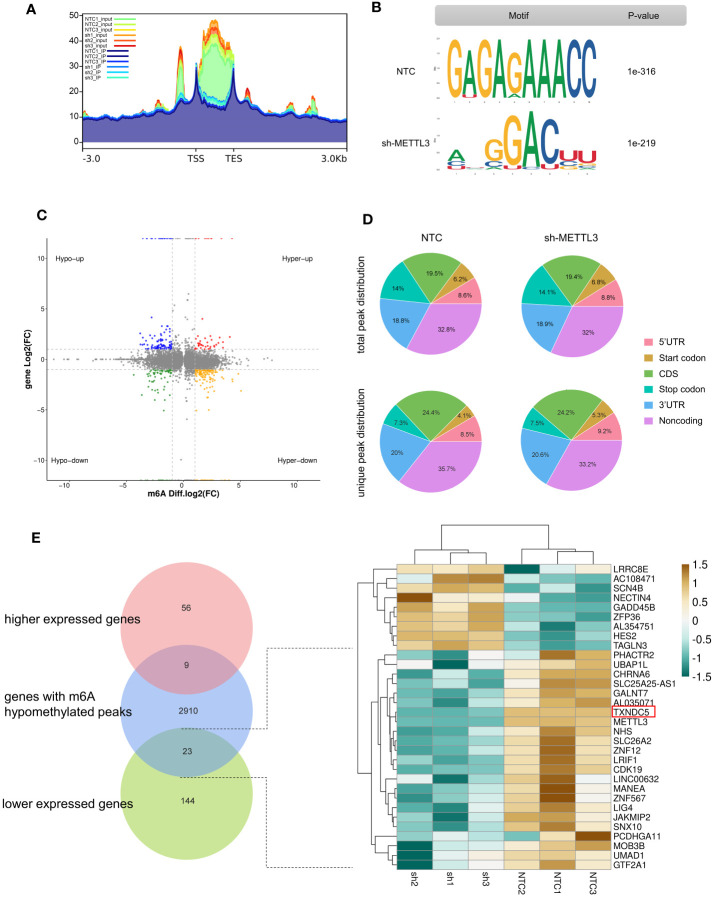
Effects of METTL3 are mediated *via* m^6^A methylation. **(A)** Metagene analysis showing different m^6^A peak distribution between NTC and METTL3 knockdown groups. **(B)** “RRACH” as the consensus motif identified by HOMER with m^6^A peaks in A375 cells with or without METTL3 knockdown. **(C)** Distribution of genes showing significant changes in both m^6^A level (log2 fold-change) and gene expression level (log2 fold-change, p < 0.05) in NTCs and sh-METTL3 A375 cells. **(D)** Proportion of m^6^A peak distribution in 5′-UTR, start codon, noncoding, coding sequence, stop codon, and 3′-UTR regions in the entire set of RNA transcripts; the total and unique m^6^A peaks in both groups are shown. **(E)** Venn diagram and heatmap illustrating genes as potential targets of METTL3 identified by MeRIP-seq and RNA-seq.

As the core methyltransferase, METTL3 regulates m^6^A modification, as suggested by the decrease in m^6^A-modified RNA under METTL3 depletion. To clarify this observation, we performed methylated RNA immunoprecipitation sequencing (MeRIP-seq) to map the m^6^A modification in sh-METTL3 and NTC cells (**Figure 6A**). Raw sequencing reads were processed to discard adaptor sequences and low-quality bases using the Trimmomatic v0.3^6^ tool. The m^6^A consensus RRACH motif exhibited high enrichment in sh-METTL3 and NTC cells (**Figure 6B**). Importantly, in shMETTL3 (sh) cells, nearly 82% (65 of 80) of differentially m^6^A-modified transcripts were downregulated (fold-change ≥2 and p < 0.05) (**Figure 6C**). Read distribution analysis showed that the NTC and METTL3 knockdown groups has a similar pattern for the total m^6^A distribution, and reads from m^6^A-IP samples were highly accumulated around the noncoding area, coding sequence, and within 3′-untranslated regions (3′-UTRs) under all experimental conditions (**Figure 6D**). By integrating the m^6^A input RNA-seq dataset and 232 DEGs previously obtained, 31 genes were found to be affected by the methylation of m^6^A catalyzed by METTL3, with 23 genes downregulated and 9 genes upregulated, indicating their potential as METTL3 targets (**Figure 6E**).

### METTL3 Targeted TXNDC5 in Melanoma Regulation

**Figure 7:**
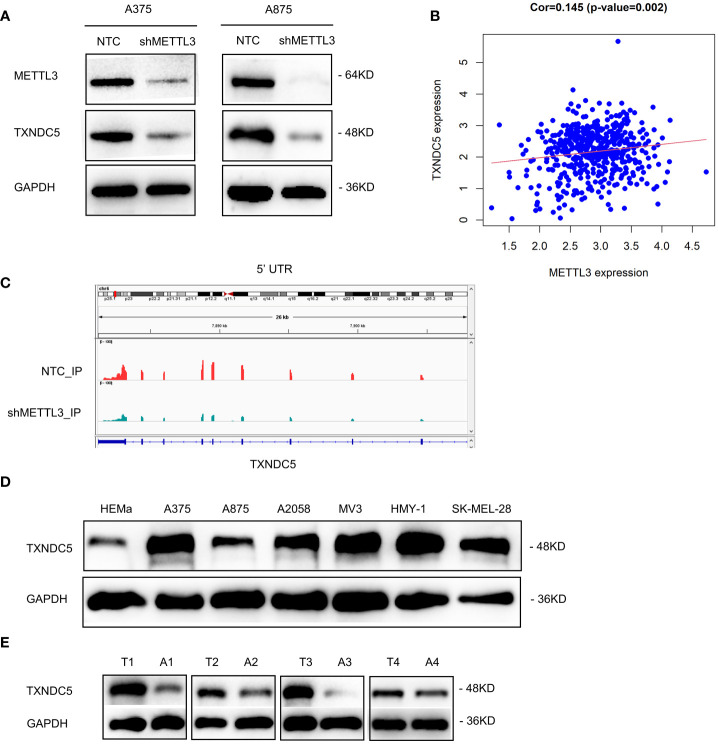
METTL3 targeted TXNDC5 in melanoma regulation. **(A)** METTL3 knockdown downregulated the protein level of TXNDC5. **(B)** METTL3 expression was positively correlated with TXNDC5 expression in melanoma in TCGA. **(C)** database.m^6^A abundance on TXNDC5 mRNA was plotted by the IGV. METTL3 knockdown diminishes m^6^A modification of TXNDC5 mRNA in A375 cells. **(D, E)** TXNDC5 was significantly upregulated in melanoma cell lines and samples.

To further screening the target genes of METTL3, Western blotting was performed to preliminary verify the potential targets from MeRIP-seq and RNA-seq. Intriguingly, consistent with the gene expression data, knockdown of METTL3 downregulated TXNDC5 at both the mRNA and protein levels (**Figure 7A**). According to The Cancer Genome Atlas (TCGA) melanoma dataset, intratumoral TXNDC5 expression was positively correlated with METTL3 expression (**Figure 7B**). Moreover, in A375 NTC/shMETTL3 cells, peak calling analysis distinguished an m^6^A peak enrichment in the 5′-UTR of TXNDC5 mRNA that was diminished upon METTL3 knockdown (**Figure 7C**). Meanwhile, we validated TXNDC5 higher expression in melanoma cell lines and melanoma tissues compared with HEMa and adjacent normal tissues, respectively (**Figures 7D, E**). Taken together, TXNDC5 may be the downstream target of METTL3-m^6^A in melanoma and may play an important role in melanoma carcinogenesis.

## Discussion

Here, we observed increased m^6^A modification levels in acral melanoma tumor tissues. To our knowledge, this is the first report of m^6^A dysregulation in primary acral melanoma tissues. Abnormal expression of the core methyltransferase METTL3 was suggested as an underlying cause of this phenomenon. Moreover, high METTL3 mRNA expression was significantly associated with clinical staging in melanoma. No melanoma cases in this study received any drug treatment. Thus, identification in their specimens from primary sites suggested specific changes in m^6^A modification and methyltransferase METTL3 levels occurring in the early stages of acral melanoma pathogenesis. As such, our findings support the use of METTL3 as a biomarker of acral melanoma initiation. Previously, the critical role of METTL3 mainly reflected in the great impact on cell proliferation, growth, survival, invasion, and angiogenesis (39–42). However, the regulation mechanism of METTL3 through m^6^A modification is diverse and elusive. In our study, METTL3 was found to promote melanoma development by enhancing cell growth and invasion *in vivo* and *in vitro*. RNA-seq analysis under METTL3 knockdown revealed DEGs involved in the cell cycle, apoptosis process, immune response, cell adhesion, and defense response, supporting the tumor-promoting effect of METTL3, and reflected that the transcription regulation underlies the tumor-promotor role METTL3.

Recent findings regarding the function of m^6^A in cancer progression are controversial. Ma et al. reported that the N^6^-adenosine methyltransferase METTL14 was significantly downregulated in hepatocellular carcinoma, in parallel with an overall decrease in m^6^A-modified RNA, which in turn affected the tumor suppressor miR-126, ultimately promoting the metastasis and invasion of hepatocellular carcinoma (23). In contrast, Chen et al. found that overexpression of the methyltransferase METTL3 was related to the poor prognosis of hepatocellular carcinoma, promoting its proliferation, migration, and lung metastasis through the m^6^A-dependent YTHDF2-SOCS2 pathway (43). Previous studies of m^6^A levels and METTL3 in melanoma have also yielded inconsistent results. For example, m^6^A levels were increased in addition to METTL3, and demethylase ALKBH5 was found to be significantly down- and upregulated, respectively, in ocular melanoma tumor tissues and cell lines (44). In contrast, a study of uveal melanoma showed the opposite results, reporting upregulation of both m^6^A modifications and METTL3 and highlighting their role as cancer-promoting factors (45). Yang et al. found that FTO expression was elevated in cutaneous melanoma tissues, with FTO knockdown inhibiting cell proliferation, migration, and metastasis. Knockdown of METTL3/METTL14 in Mel624 cells had the opposite effects (46). The discrepancies between these previous findings underscore the complexity of m^6^A modification and its regulatory enzymes in human cancers. This may be because of the involvement different reader proteins that facilitate m^6^A modification-associated signaling, different cellular functions regulated by the target genes, and distinct mRNA regions of m^6^A distribution. Further studies are required to evaluate these controversial results. This also indicates the great significance to explore the downstream targets of METTL3.

TXNDC5 is a copious enzyme in fibroblast-enriched endoplasmic reticulum (47). As a critical member of the protein disulfide isomerase (PDI) family, the aberrant overexpression of TXNDC5 protein has been investigated in multiple human tumors, including esophageal squamous cell carcinoma (48), cervical cancer (49), lung cancer (50), and hepatocellular carcinoma (51). In a previous study, multiple mechanisms have been described whereby TXNDC5 contributes to cancer development, which is mainly evident in promoting tumor survival and growth in hypoxia environment, altering tumor microenvironment to assists tumor cell metastasis and invasion, and interacting with plasma membrane receptors to control oncogenic cellular response (52). Our study conducted MeRIP-seq to explore the potential mRNAs regulated by METTL3 and found a series of genes whose transcripts were subjected to METTL3-mediated m^6^A modification. We identified that TXNDC5 was decreased in RNA level and m^6^A level after METTL3 knockdown. Similarly, the enhanced protein expression of TXNDC5 was also confirmed in melanoma tissues and cell lines. Analysis of the underlying mechanism of METTL3 suggested that it affected melanoma cell growth and invasion by regulating TXNDC5. These results suggest that METTL3 may affect the tumorigenesis of melanoma by modulating transcripts of TXNDC5 in an m^6^A-dependent manner.

Few studies have explored the functions of METTL3 inside melanoma cells. Wu et al. found that UCK2 overexpression was regulated by m^6^A-METTL3 axis in melanoma metastasis (53); Chang et al. proposed that miR-302a-3p targets and suppresses the expression of METTL3 to inhibit melanoma cell progression (54). In accordance with this, elevated METTL3 expression was detected in human melanoma cell lines, which led to increased m^6^A activity, colony formation, and invasion of melanoma cells through MMP2 and N-cadherin accumulation (55). In consistence with their studies, we also identified the critical role of METTL3 in melanoma cell growth, migration, and invasion. However, our research obtained first insight into the dysregulation of m^6^A modification and METTL3 in Chinese acral melanoma tissues and revealed the clinic significance of METTL3. We also found that TXNDC5 served as a novel target of METTL3-m^6^A axis in acral melanoma.

Regrettably, due to the limited AM population, the risk prediction role of METTL3 in the progression of AM may not be fully revealed in the current study; more advanced patients will be incorporate in our future studies. In conclusion, we revealed the critical role of METTL3-mediated m6A modification in acral melanoma progression; increased m6A level was confirmed in acral melanoma samples. METTL3 was essential for acral melanoma carcinogenesis and progression, as featured by promoting cancer cell proliferation, migration, and invasion. Importantly, we uncovered that METTL3 affects melanoma progression by targeting m6A-TXNDC5 mRNA, and it is hoped that these findings will contribute to a potential therapeutic strategy in acral melanoma.

## Data Availability Statement

The datasets presented in this study can be found in online repositories. The names of the repository/repositories and accession number(s) can be found below: https://www.ncbi.nlm.nih.gov/geo/query/acc.cgi?acc=GSE183967

## Ethics Statement

The studies involving human participants were reviewed and approved by Ethics Committee of the Institute of Dermatology at the Chinese Academy of Medical Sciences. The patients/participants provided their written informed consent to participate in this study.

## Author Contributions

Conceptualization: JS and YW. Data curation: ZY, MC, and AH. Formal analysis: ZY, QZ, and MC. Funding acquisition: JS and YW. Investigation: ZY, QZ, and MC. Methodology: QZ and ZJ. Project administration: JS and YW. Resources: JS, YW, ZJ, GZ, QW, LZ, and FF. Software: QZ and AH. Supervision: JS, YW, and QW. Validation: MC, AH, and QZ. Visualization: ZY and MC. Writing—original draft preparation: ZY and AH. Writing—review and editing: YW and JS.

## Funding

This work was supported by the National Natural Science Foundation of China (grant number 81872216), PUMC Youth Fund (grant number 3332020105), and Nanjing Incubation Program for National Clinical Research Center (grant number 2019060001).

## Conflict of Interest

The authors declare that the research was conducted in the absence of any commercial or financial relationships that could be construed as a potential conflict of interest.

## Publisher’s Note

All claims expressed in this article are solely those of the authors and do not necessarily represent those of their affiliated organizations, or those of the publisher, the editors and the reviewers. Any product that may be evaluated in this article, or claim that may be made by its manufacturer, is not guaranteed or endorsed by the publisher.

## Abbreviations

AM, acral melanoma; HEMa, human epidermal melanocytes; m^6^A, N^6^-methyladenosine; METTL3, methyltransferase like 3; TXNDC5, thioredoxin domain containing protein 5; qRT-PCR, quantitative real-time reverse transcriptase PCR; NTC, non-targeted control; IHC, immunohistochemistry; DEG, differentially expressed gene; GAPDH, glyceraldehyde 3-phosphate dehydrogenase; MeRIP-Seq, methylated RNA immunoprecipitation sequencing; RNA-seq, RNA sequencing; CDS, coding sequence; 3′-UTR, 3 primer untranslated region; 5′-UTR, 5 primer untranslated region.
