# Supplementary material for: m6A Methyltransferase METTL3 Promotes the Progression of Primary Acral Melanoma via Mediating TXNDC5 Methylation
Source: Front Oncol. 2022 Jan 18;11:770325. doi: 10.3389/fonc.2021.770325 (PMC8804213; doi:10.3389/fonc.2021.770325)
Supplement: Supplementary file 1 [file DataSheet_1.pdf]

Supplementary Material

a

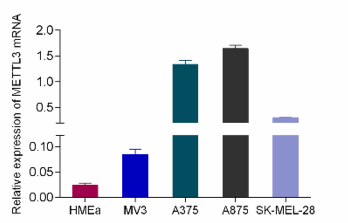

Supplementary figure1 (a) qRT-PCR analysis revealed greatly upregulated METTL3 mRNA levels in melanoma cells compared to HEMa.

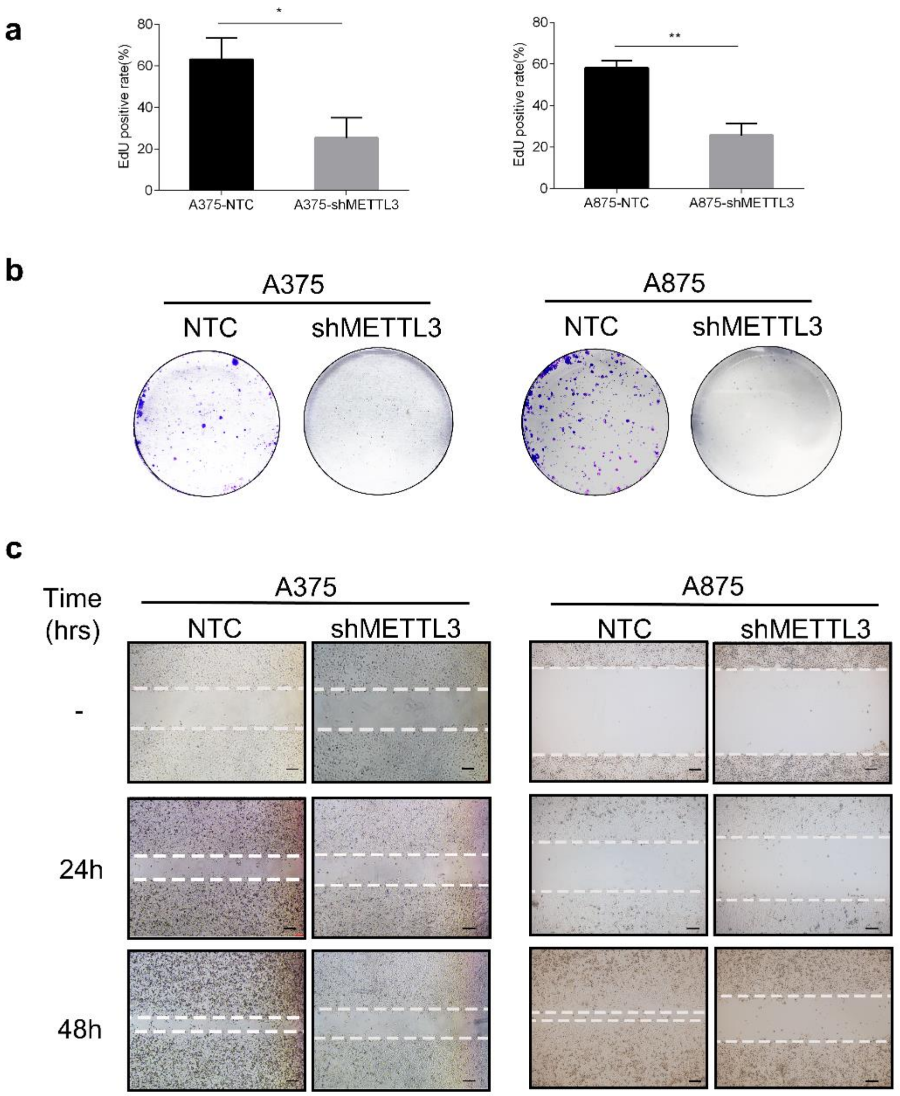

Supplementary figure2 (a) Analysis of EdU positive cells showed in column diagram. (b) The typical images of plate colony formation showed that knockdown of METTL3 significantly decreased the cloning number of A375 and A875 cells compared with control groups. (C) Representative images of scratch wound healing assay at 0, 24, 48 hours.

Supplementary T able S1. Primers used in this study

Gene	Primer sequence (5’-3’)	
**qRT-PCR**		
METTL3	**F:** CAAGCTGCACTTCAGACGAA	**R:** GCTTGGCGTGTGGTCTTT
GAPDH	**F:** GAGTCAACGGATTTGGTCGT	**R:** TTGATTTTGGAGGGATCTCG

Supplementary Table S2. 232 differentially expressed genes

Gene_name	NTC	shMETTL3	log2(fc)	P value	regulation
METTL3	7.707098	1.716208	-2.17	2.65E-60	down
AC138696	1.453246	0	-inf	4.08E-58	down
GDF15	13.06903	39.32861	1.59	1.64E-35	up
ZFP36	5.179944	15.42439	1.57	1.10E-29	up
AP002990	18.25795	205.8697	3.50	4.39E-22	up
EIF4EBP2	9.025544	3.930048	-1.20	3.55E-20	down
IPO4	2.294439	19.05008	3.05	7.68E-20	up
CXCL1	3.145119	9.992273	1.67	8.86E-18	up
TXNDC5	33.9036	16.05436	-1.08	3.04E-16	down
EIF5	4.78368	1.834613	-1.38	7.50E-15	down
CDKN1A	86.81642	179.3885	1.05	1.91E-14	up
CBFB	6.170556	2.546554	-1.28	2.99E-13	down
MIR1244-2	26.0195	2.754928	-3.24	1.29E-12	down
CXCL8	1.910798	5.107847	1.42	3.00E-12	up
KLF9	41.45955	20.15657	-1.04	5.72E-11	down
TUT1	18.11516	8.03066	-1.17	6.58E-11	down
NFATC2	23.37593	11.15283	-1.07	1.46E-10	down
ARC	0.62679	1.680116	1.42	1.67E-10	up

BIVM-ERCC5	0	1.482725	inf	2.41E-10	up
SLC26A2	50.36945	24.93554	-1.01	2.80E-10	down
IL6ST	6.490429	3.059799	-1.08	7.02E-10	down
FN1	14.98648	7.443563	-1.01	7.83E-10	down
AC008763	12.3624	0	-inf	1.63E-09	down
GADD45B	2.03119	4.39088	1.11	2.69E-09	up
DCT	3.259335	1.30677	-1.32	3.39E-09	down
AC010422	0.958629	0	-inf	3.72E-09	down
RASSF8	5.55708	2.708203	-1.04	1.58E-08	down
PCSK2	1.89316	0.795764	-1.25	2.35E-08	down
SERPINE1	0.16203	0.502306	1.63	2.54E-08	up
RNF150	2.536392	1.239358	-1.03	2.55E-08	down
NRIP1	3.792154	1.742646	-1.12	3.59E-08	down
RPSAP26	1.621192	0.240713	-2.75	3.64E-08	down
DIXDC1	1.784804	0.796141	-1.16	5.01E-08	down
IL1RAP	2.429307	1.111019	-1.13	5.22E-08	down
DERPC	23.17884	9.782249	-1.24	5.90E-08	down
SNX10	4.200862	1.724443	-1.28	6.21E-08	down
TMEM19	2.382926	1.061661	-1.17	6.81E-08	down
RN7SKP230	2.991431	32.7941	3.45	1.56E-07	up
HES2	0.237982	0.57922	1.28	2.02E-07	up
ERO1B	5.156873	2.506627	-1.04	2.22E-07	down
TREX1	0.714062	4.015144	2.49	3.30E-07	up
TRIM34	0.510258	0.036159	-3.82	3.42E-07	down
NEK7	4.087935	1.998166	-1.03	4.10E-07	down
NR2C2	2.844427	1.159413	-1.29	7.76E-07	down
RAB43	0.203002	2.296151	3.50	7.94E-07	up
MATR3	11.83066	5.226761	-1.18	8.16E-07	down
CXCL2	0.437223	1.256945	1.52	1.10E-06	up
KIAA1143	1.894198	0.813011	-1.22	1.39E-06	down
AP000350	0.405932	0.12516	-1.70	1.80E-06	down

LINC00475	0.111317	0.311562	1.48	2.39E-06	up
FAM20A	2.334177	1.137809	-1.04	3.17E-06	down
SLC7A2	1.20582	0.53271	-1.18	3.39E-06	down
PTPRZ1	0.956816	0.386586	-1.31	3.54E-06	down
INPP5D	0.186998	0.432119	1.21	4.28E-06	up
KRAS	3.060521	1.522289	-1.01	6.80E-06	down
BCLAF1P2	0.510454	0.07422	-2.78	7.04E-06	down
TAGLN3	1.106836	2.214279	1.00	7.29E-06	up
TFAM	2.064194	0.90639	-1.19	1.05E-05	down
MT-ND2	5.407589	2.258381	-1.26	1.22E-05	down
CXCL3	0.150298	0.433561	1.53	1.58E-05	up
AC010336	2.821873	1.006159	-1.49	1.65E-05	down
SNORA64	14.07457	39.09995	1.47	1.79E-05	up
TXNIP	75.20681	160.7235	1.10	1.80E-05	up
RPL7AP66	43.72063	3.580011	-3.61	2.16E-05	down
MIR378D2HG	0.932718	2.072457	1.15	2.46E-05	up
FAM172A	1.6777	0.787731	-1.09	2.47E-05	down
CDK19	1.074981	0.505677	-1.09	2.56E-05	down
CISH	0.224339	0.465037	1.05	2.59E-05	up
SCN4B	0.061089	0.151725	1.31	2.94E-05	up
DDIAS	1.284295	0.585057	-1.13	3.61E-05	down
EIF4BP6	10.68465	3.493797	-1.61	4.14E-05	down
ZNF644	1.366659	0.63645	-1.10	4.66E-05	down
AC105137	0.900178	2.034209	1.18	4.84E-05	up
RGS8	1.050379	0.504132	-1.06	6.02E-05	down
MT-ND1	6.546642	3.044146	-1.10	7.28E-05	down
C13orf46	1.702024	0.802564	-1.08	7.29E-05	down
WDR74	21.97766	279.5165	3.67	7.51E-05	up
SAMD9	0.565416	0.2196	-1.36	8.41E-05	down
GALNT7	0.762485	0.354563	-1.10	1.06E-04	down
PLEKHH1	1.050656	0.524349	-1.00	1.06E-04	down
PDE3A	0.462098	0.220621	-1.07	1.34E-04	down

EEF1A1P13	5.000402	10.20692	1.03	1.35E-04	up
RBAK	0.500778	0.211898	-1.24	1.49E-04	down
AL035071	1.045845	0.413224	-1.34	1.71E-04	down
PDE7B	0.394628	0.155292	-1.35	1.76E-04	down
AL627171	6.560216	0.449872	-3.87	1.82E-04	down
ELMO3	0.874938	0.364328	-1.26	1.92E-04	down
NECTIN4	0.118736	0.333411	1.49	1.97E-04	up
AL356515	1.173798	2.493982	1.09	1.98E-04	up
RNU11	5.582282	54.61524	3.29	2.07E-04	up
PPIAP29	10.02001	48.82504	2.28	2.16E-04	up
PCDHGA11	0.848974	0.072029	-3.56	2.40E-04	down
AL137798	0.619661	1.567752	1.34	2.87E-04	up
PCDHGB2	1.444148	0.225485	-2.68	2.90E-04	down
AL596244	0.346008	0.134047	-1.37	3.21E-04	down
AL355385	0.18589	0.578363	1.64	3.28E-04	up
RPS26P8	2.400305	19.59962	3.03	3.37E-04	up
C7orf31	0.491246	0.206021	-1.25	3.74E-04	down
AL354751	0.203253	0.433752	1.09	3.80E-04	up
IL32	0.115905	0.259665	1.16	3.87E-04	up
NHS	0.163034	0.065416	-1.32	3.99E-04	down
AC073091	0.924525	0.404284	-1.19	4.01E-04	down
RNMT	2.512878	0.973083	-1.37	4.08E-04	down
AL450405	29.61583	61.0258	1.04	4.18E-04	up
LINC00632	0.895098	0.444811	-1.01	4.28E-04	down
TTN	0.0091	0.002652	-1.78	4.37E-04	down
AC020907	0.293701	0.664526	1.18	4.37E-04	up
AL136295	35.11542	13.50363	-1.38	4.64E-04	down
CARNMT1	1.061792	0.516323	-1.04	4.67E-04	down
PIK3R1	1.46966	0.713292	-1.04	4.92E-04	down
PARD6B	0.988111	0.440163	-1.17	5.45E-04	down
RESF1	0.613214	0.271383	-1.18	5.61E-04	down

CLEC18A	0.180493	0.071797	-1.33	5.66E-04	down
TBCEL-TECTA	0.515566	0.10652	-2.28	6.30E-04	down
ZNF253	0.393859	0.163424	-1.27	6.37E-04	down
C19orf81	0.613116	1.295309	1.08	6.64E-04	up
RMI1	1.575991	0.768228	-1.04	6.72E-04	down
CCDC144A	0.029283	0.066316	1.18	7.23E-04	up
OR7E38P	1.488106	7.520818	2.34	7.33E-04	up
SGO2	0.635703	0.275197	-1.21	7.66E-04	down
ZNF12	0.961364	0.474753	-1.02	8.07E-04	down
FAM111B	0.397441	0.147268	-1.43	8.57E-04	down
PCDHGC5	0.118028	0.720496	2.61	9.24E-04	up
LIG4	1.040216	0.491125	-1.08	9.43E-04	down
AP003900	3.404096	1.156741	-1.56	9.67E-04	down
LRIF1	1.04254	0.505243	-1.05	9.87E-04	down
AL137782	0.262985	0.704509	1.42	1.03E-03	up
ERCC5	2.23827	0.837342	-1.42	1.11E-03	down
PCSK6	0.260161	0.116038	-1.16	1.12E-03	down
AL590004	0.391566	0.178047	-1.14	1.19E-03	down
TRAPPC5	1.629445	4.617087	1.50	1.25E-03	up
MUC15	0.391213	0.150022	-1.38	1.26E-03	down
ZNF480	0.29446	0.124698	-1.24	1.28E-03	down
AC108471	0.11394	0.237763	1.06	1.28E-03	up
PRKAA2	0.375291	0.182702	-1.04	1.31E-03	down
NPHP3-ACAD11	1.168407	0.210959	-2.47	1.32E-03	down
SLC24A3	0.31801	0.133324	-1.25	1.32E-03	down
ZNF813	0.204877	0.082469	-1.31	1.32E-03	down
ETAA1	0.562125	0.280539	-1.00	1.47E-03	down
LRRC8E	2.12314	4.470154	1.07	1.47E-03	up
SNORA71A	3.872547	8.827575	1.19	1.48E-03	up
PPP3CB-AS1	0.179448	0.069131	-1.38	1.51E-03	down
MAP9	0.43519	0.203148	-1.10	1.54E-03	down
SNX18P3	0.537741	0.205811	-1.39	1.55E-03	down

PCDHGB1	0.524083	2.038169	1.96	1.64E-03	up
LINC01843	0.379766	0.762657	1.01	1.66E-03	up
AC006064	1.751875	0.023552	-6.22	1.74E-03	down
ACSBG1	0.268788	0.103361	-1.38	1.98E-03	down
GOLGA2P5	0.081819	0.033878	-1.27	2.04E-03	down
PTCH2	0.219859	0.073737	-1.58	2.04E-03	down
KRT14	0.713412	1.529664	1.10	2.05E-03	up
ATAD3C	0.117828	0.258064	1.13	2.07E-03	up
ESPNP	0.344248	0.870717	1.34	2.15E-03	up
FOXR2	0.52753	0.245091	-1.11	2.33E-03	down
GTF2A1	2.478302	1.162454	-1.09	2.37E-03	down
KIF18A	1.128824	0.561308	-1.01	2.51E-03	down
PCDHGA1	0.770522	0.192992	-2.00	2.61E-03	down
ZNF347	0.289998	0.143504	-1.01	2.71E-03	down
IFNAR2	2.240705	1.037137	-1.11	2.79E-03	down
SLC7A5P2	1.940188	4.098981	1.08	2.96E-03	up
FTH1P2	6.820133	1.92608	-1.82	2.97E-03	down
ADRA2A	0.236969	0.096221	-1.30	3.07E-03	down
AL133352	0.264808	1.689437	2.67	3.50E-03	up
BIVM	2.332731	1.138901	-1.03	4.03E-03	down
AC005831	1.410012	0.702002	-1.01	4.05E-03	down
ZNF845	0.319446	0.148648	-1.10	4.14E-03	down
MANEA	0.450028	0.206051	-1.13	4.15E-03	down
LINC00565	0.190247	0.072438	-1.39	4.21E-03	down
ZNF443	0.436522	0.202062	-1.11	4.31E-03	down
CHRNA6	0.714339	0.344809	-1.05	4.59E-03	down
RPS3AP47	12.16324	5.184226	-1.23	4.62E-03	down
AC092718	3.021628	1.472874	-1.04	4.77E-03	down
SHLD3	0.558076	0.176226	-1.66	5.20E-03	down
MST1L	0.274264	0.652297	1.25	5.31E-03	up
ZNF180	0.289177	0.136597	-1.08	5.34E-03	down

SENP7	0.509196	0.245838	-1.05	5.42E-03	down
AL049629	5.768578	2.120774	-1.44	5.53E-03	down
ACTA1	0.62044	0.133781	-2.21	5.74E-03	down
BX284668	0.436559	1.057864	1.28	5.80E-03	up
LINC00641	0.200961	0.079112	-1.34	6.29E-03	down
AL357079	0.444505	0.20685	-1.10	6.33E-03	down
ZNF260	0.484584	0.232753	-1.06	6.62E-03	down
AC016026	8.658369	3.642642	-1.25	6.63E-03	down
H2BS1	9.302814	1.750259	-2.41	6.84E-03	down
AC007406	0.302996	0.136931	-1.15	6.92E-03	down
ZNF597	0.346263	0.16949	-1.03	7.11E-03	down
ZNF713	0.139245	0.064427	-1.11	7.22E-03	down
JAKMIP2	0.109821	0.050416	-1.12	7.37E-03	down
PHACTR2	0.49912	0.234156	-1.09	8.32E-03	down
AL449403	0.471359	0.226187	-1.06	8.88E-03	down
PCDHGA4	0.450546	0.140948	-1.68	8.96E-03	down
SLC25A25-AS1	0.267117	0.132565	-1.01	8.98E-03	down
CA12	0.102763	0.04478	-1.20	9.50E-03	down
ZNF92	0.359464	0.168951	-1.09	9.60E-03	down
RADX	0.319461	0.155929	-1.03	9.80E-03	down
LINC00622	0.962522	0.436572	-1.14	1.06E-02	down
CAPN3	0.067222	0.027239	-1.30	1.13E-02	down
AL049839	154.7535	64.46962	-1.26	1.14E-02	down
CYP4F26P	0.240214	0.10061	-1.26	1.18E-02	down
SASS6	0.901012	0.448118	-1.01	1.20E-02	down
ABHD14A-ACY1	0.809781	0.308317	-1.39	1.25E-02	down
PABPC4L	0.260724	0.128798	-1.02	1.36E-02	down
ZNF658B	0.225454	0.094306	-1.26	1.49E-02	down
ZNF567	0.22855	0.109621	-1.06	1.54E-02	down
LINC00997	0.326812	0.157755	-1.05	1.59E-02	down
KCTD21	0.682219	1.410602	1.05	1.61E-02	up
AL669830	1.349753	0.581206	-1.22	1.77E-02	down

MOB3B	0.182518	0.090929	-1.01	1.91E-02	down
ZBED9	0.13794	0.067435	-1.03	2.18E-02	down
UMAD1	1.481344	0.630264	-1.23	2.24E-02	down
CU633967	0.217661	0.06988	-1.64	2.35E-02	down
AC090498	13.19731	29.08857	1.14	2.36E-02	up
RNY3P1	36.91445	99.21236	1.43	2.49E-02	up
PPAN-P2RY11	5.279035	0.544677	-3.28	2.56E-02	down
RPL7P23	4.658032	1.876193	-1.31	2.62E-02	down
Y_RNA	36.60553	98.31996	1.43	2.64E-02	up
MAGEA10-MAGEA5	0.940854	0.465163	-1.02	2.74E-02	down
RPSAP54	3.97	10.04619	1.34	2.76E-02	up
H1-12P	2.791471	0.878111	-1.67	3.03E-02	down
RN7SKP255	41.85762	20.24913	-1.05	3.25E-02	down
KCNJ10	0.189673	0.090338	-1.07	3.27E-02	down
MYH2	0.152997	0.026581	-2.53	3.30E-02	down
TIMM23B-AGAP6	0.645497	0.21851	-1.56	3.36E-02	down
NUDT4B	1.112219	2.594792	1.22	3.37E-02	up
UBAP1L	0.11284	0.05349	-1.08	3.93E-02	down
AC004593	0.171391	0.034202	-2.33	4.14E-02	down
BCAP29	1.505927	0.68155	-1.14	4.14E-02	down
CALML4	0.328631	0.130604	-1.33	4.20E-02	down
NUDT4P2	2.023142	4.228267	1.06	4.41E-02	up
SLC16A2	0.203838	0.096612	-1.08	4.46E-02	down
Z83844	1.446222	3.484763	1.27	4.77E-02	up
UCHL3	1.706561	0.714516	-1.26	4.95E-02	down
